# Complete Sequences of Two Plasmids Found in a Brazilian Bacillus thuringiensis Serovar israelensis Strain

**DOI:** 10.1128/MRA.00051-19

**Published:** 2019-02-28

**Authors:** Fabrício S. Campos, Fernando B. Cerqueira, Gil R. Santos, Eliseu J. G. Pereira, Roberto F. T. Corrêia, Alex S. R. Cangussu, Fernando L. Melo, Bergmann M. Ribeiro, Raimundo W. S. Aguiar

**Affiliations:** a Laboratory of Bioinformatics & Biotechnology, Campus de Gurupi, Federal University of Tocantins, Gurupi, Tocantins, Brazil; b Plant Production Graduate Program, Campus de Gurupi, Federal University of Tocantins, Gurupi, Tocantins, Brazil; c Entomology Department, Federal University of Viçosa, Viçosa, Minas Gerais, Brazil; d Biotechnology Graduate Program, Campus de Gurupi, Federal University of Tocantins, Gurupi, Tocantins, Brazil; e Cell Biology Department, University of Brasilia, Brasilia, Brazil; f Biotechnology and Plant Production Graduate Program, Campus de Gurupi, Federal University of Tocantins, Gurupi, Tocantins, Brazil; Loyola University Chicago

## Abstract

Plasmids play a crucial role in the evolution of bacterial genomes by mediating horizontal gene transfer. In this work, we sequenced two plasmids found in a Brazilian Bacillus thuringiensis serovar israelensis strain which showed 100% nucleotide identities with Bacillus thuringiensis serovar kurstaki plasmids.

## ANNOUNCEMENT

Horizontal gene transfer mediated by plasmids is an important bacterial evolutionary driving force. Plasmids harbor genes involved in niche-specific processes and are important for bacterial adaptation to changing environmental conditions (1). In Bacillus thuringiensis insecticidal toxin genes (*cry*) reside on large self-transmissible plasmids, and individual B. thuringiensis strains can harbor a diverse range of plasmids that can vary in number and size from around 2 to 200 kb (2). B. thuringiensis small plasmids generally replicate by using the rolling-circle replication mechanism with single-stranded DNA intermediates. In addition, B. thuringiensis small plasmids usually have high copy numbers and no known specific functions, which is why they are called “cryptic” (3).

In this report, we were able to determine the complete DNA sequence of two plasmids from a Brazilian B. thuringiensis serovar israelensis strain (Bti-UFT6.51) which was isolated from soil samples (4) collected in the state of Tocantins, Brazil, and was shown to have high toxicity toward lepidopteran larvae (data not shown). These two new plasmids were identified by sequencing the full B. thuringiensis serovar israelensis strain genome and were designated pBtiUFT6.51.1 and pBtiUFT6.51.2. The new plasmid sequences showed high nucleotide sequence identity with two B. thuringiensis serovar kurstaki plasmids, pBMB74 (GenBank accession number NZ_CP004874) and unnamed11 (NZ_CP010009), respectively.

Total genomic DNA was extracted and purified using the Wizard genomic DNA puriﬁcation kit (Promega) according to the manufacturer’s instructions. The genomic DNA library preparation procedure followed the Nextera DNA sample preparation guide (Illumina, USA). The total genome was sequenced using the MiSeq platform (Illumina) with paired-end applications (2 × 150 bp) at the Catholic University of Brasília, Brazil. A total of 5,502,614 paired-end reads were generated at a read length of 150 bp. The quality read libraries were analyzed using the software FastQC version 0.11.3, and sequence reads were quality trimmed with a minimum Phred quality of >20. DNA sequence assembly using the map to reference function in Geneious version 9.1.8 was used (5). For this, plasmids pBMB74 (GenBank accession number NZ_CP004874) and unnamed11 (NZ_CP010009) were used separately as the reference sequence. The pBtiUFT6.51.1 plasmid sequence was assembled into a contig of 74,336 bp, from a total of 142,766 reads, with an average length of 77 bp, i.e., a coverage of 147 times. The pBtiUFT6.51.2 plasmid sequence was assembled into a contig of 8,279 bp, from a total of 47,185 reads, with an average length of 77 bp, i.e., a coverage of 438 times. Thereafter, we did a multiple sequence alignment between plasmids pBMB74 and pBtiUFT6.51.1 and plasmids unnamed11 and pBtiUFT6.51.2 using the MAFFT alignment plugin with default settings in Geneious version 9.1.8 (5). The aligned plasmids presented 100% nucleotide identity.

B. thuringiensis strains contain a set of self-replicating, transmissible extrachromosomal DNA molecules or plasmids, which vary in number and size in different strains (6). The widespread occurrence of self-transmissible plasmids in B. thuringiensis strains suggests that conjugation may be an important means of plasmid dissemination in *Bacillus* populations in nature. Thus, the high similarity between plasmids (3) from different regions and countries indicates an evolutionary relationship among *Bacillus* species.

### Data availability.

The GenBank accession numbers for the plasmids in this study are MG710485 (pBtiUFT6.51.1) and MG710483 (pBtiUFT6.51.2). The Sequence Read Archive (SRA) accession number is SRX5193168.
